# Vessel-Specific Quantitative Plaque Characteristics on Coronary CT Angiography for Prediction of FFR-Defined Myocardial Ischemia

**DOI:** 10.3390/diagnostics16213571

**Published:** 2026-10-29

**Authors:** Shuang Li, Tian Ma, Anqi Yang, Yinghao Xu, Yi He

**Affiliations:** 1 Department of Radiology, Beijing Friendship Hospital, Capital Medical University, No. 95 Yongan Road, Beijing 100050, China; lxs3926@163.com (S.L.);; 2 Canon Medical Systems (China) Co., Ltd., Building 205, Yard No. A10, Jiuxianqiao North Road, Beijing 100015, China

**Keywords:** myocardial ischemia, coronary computed tomography angiography, fractional flow reserve, plaque quantification

## Abstract

**Objectives**: To determine whether quantitative coronary computed tomography angiography (CCTA) plaque characteristics associated with fractional flow reserve (FFR)-defined ischemia vary among coronary arteries and whether they improve prediction when added to conventional clinical and stenosis information. **Methods**: This retrospective study included 204 patients and 289 vessels (161 left anterior descending [LAD], 55 left circumflex [LCX], and 73 right coronary artery [RCA] vessels) evaluated by CCTA and invasive FFR. Plaque characteristics were quantified using semi-automated software. Predictors were selected separately by vessel using the least absolute shrinkage and selection operator (LASSO) for LAD and RCA and Boruta-XGBoost for LCX. Model evaluation incorporated five-fold cross-validation and bootstrap resampling. Incremental performance was assessed by adding selected plaque features to Model 1, which comprised clinical variables and severe stenosis (≥70%). **Results**: FFR-defined ischemia (FFR ≤ 0.80) was identified in 90 LAD, 24 LCX, and 37 RCA vessels. Ischemic vessels had longer lesions, smaller minimum luminal area, greater stenosis, and higher plaque burden. Vessel-specific plaque models yielded areas under the receiver operating characteristic curve (AUCs) of 0.755 (95% CI: 0.682–0.826), 0.789 (95% CI: 0.656–0.905), and 0.775 (95% CI: 0.661–0.877) for LAD, LCX, and RCA, respectively. In a separate incremental analysis, compared with Model 1, Model 2 improved AUC in LAD (0.613 to 0.755; net reclassification improvement (NRI) = 0.736; integrated discrimination improvement (IDI) = 0.163) and RCA (0.489 to 0.767; NRI = 0.955; IDI = 0.276), with smaller gains in LCX (0.617 to 0.754; NRI = 0.651; IDI = 0.169). **Conclusions**: Quantitative CCTA plaque characteristics showed potentially vessel-dependent associations with FFR-defined ischemia. Incorporating selected plaque features into clinical and stenosis-based models improved discrimination in the LAD and RCA. These exploratory findings require validation in larger, independent cohorts.

## 1. Introduction

Coronary computed tomography angiography (CCTA) provides a noninvasive characterization of coronary atherosclerosis by depicting luminal narrowing and plaque morphology [1,2,3]. Nevertheless, the anatomical severity of a coronary lesion does not always reflect its functional significance, particularly in intermediate stenosis [4]. Assessment of plaque phenotype may therefore complement stenosis evaluation when estimating the likelihood of myocardial ischemia.

CCTA-based plaque quantification provides information beyond luminal narrowing by characterizing plaque extent and composition. Greater plaque burden, noncalcified plaque volume, and low-attenuation plaque have been linked to myocardial ischemia and adverse cardiovascular outcomes [4,5,6,7], with potential value for risk and prognostic assessment beyond conventional stenosis measures [8]. Previous studies have investigated associations between quantitative CCTA plaque characteristics and fractional flow reserve (FFR)-defined ischemia at the vessel level, including analyses across individual coronary arteries. However, the extent to which plaque characteristics provide additional predictive information beyond clinical factors and stenosis severity in different coronary territories warrants further investigation. Invasive FFR directly evaluates the physiological significance of coronary stenosis on a vessel-specific basis and serves as the reference standard for ischemia assessment [9]. Although CCTA-derived FFR (FFR-CT) enables noninvasive estimation of lesion-specific physiology [10,11], limitations in complex lesions and routine clinical settings support the continued use of invasive FFR as a functional reference [12].

The large number and intercorrelation of quantitative plaque variables pose challenges for predictive modeling. Previous CCTA studies have demonstrated applications of machine learning and radiomics to coronary inflammation, plaque characterization, and cardiovascular risk assessment [13,14]. Feature-selection approaches, including least absolute shrinkage and selection operator (LASSO) and Boruta, can help identify informative predictors while limiting model complexity [15,16]. Further investigation is warranted into whether artery-specific feature selection can identify distinct combinations of quantitative plaque characteristics and improve prediction beyond conventional clinical and anatomical variables, using invasive FFR as the reference standard.

Coronary arteries differ in anatomy, myocardial territory, and flow demand, potentially leading to vessel-specific plaque-ischemia relationships. Therefore, we investigated the association between quantitative CCTA plaque characteristics and FFR-defined ischemia across individual coronary arteries and assessed their additional predictive value beyond clinical factors and stenosis severity.

## 2. Materials and Methods

### 2.1. Patient Population

**Figure 1:**
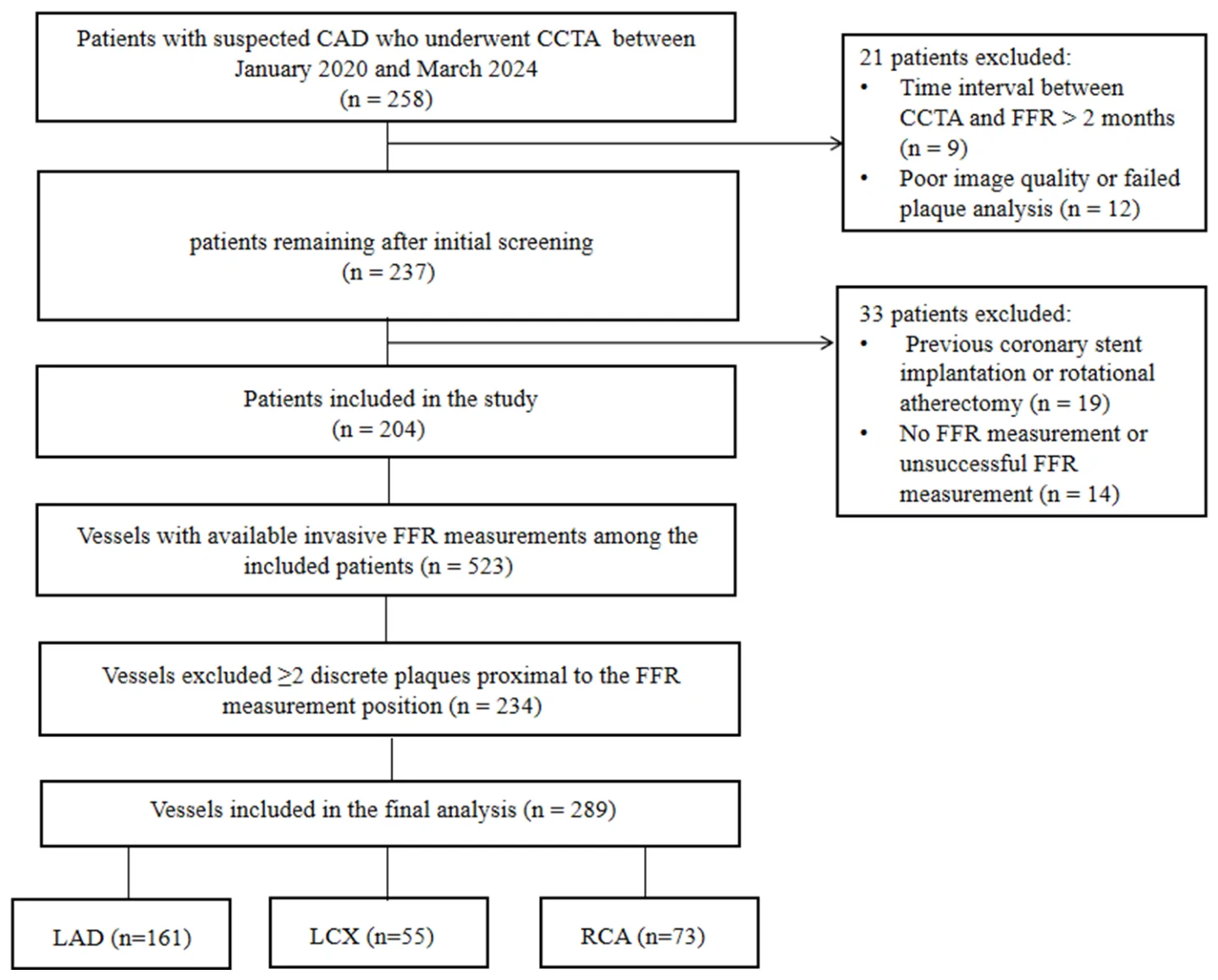
Study flow chart.

We retrospectively screened consecutive adults evaluated for suspected or established CAD at our institution from January 2020 through March 2024. Culprit lesions associated with acute myocardial infarction (MI) were excluded from the analysis. Referral to invasive coronary angiography (ICA) was based on an integrated clinical assessment of CCTA findings, clinical presentation/symptoms, and other relevant clinical information. Exclusion criteria included prior cardiac interventions (including pacemaker implantation, catheter ablation, coronary artery bypass grafting, coronary stenting, or rotational atherectomy); unsuccessful or uninterpretable FFR measurements (including coronary vasospasm, failed FFR acquisition, or incomplete physiological assessment); severe arrhythmia; an interval exceeding 2 months between CCTA and FFR; and inadequate CCTA image quality or unsuccessful plaque analysis. For vessel-level analysis, only vessels with a single identifiable CCTA plaque proximal to the FFR measurement position were included. Vessels with two or more discrete plaques proximal to the FFR measurement position were excluded to avoid ambiguity related to serial/tandem lesions. Of 258 patients screened, 204 met the study criteria, contributing 289 coronary vessels to the final dataset (Figure 1). Imaging and clinical records were de-identified and linked using study-specific identifiers. The study protocol was approved by the institutional review board (Approval No. BFHFHZS20240015), with the requirement for written informed consent waived.

### 2.2. CCTA Acquisition

CCTA examinations were performed on three multidetector CT platforms: SOMATOM Definition Flash (Siemens; *n* = 44, 21.6%), Revolution CT (GE; *n* = 63, 30.9%), and Aquilion ONE (Toshiba; *n* = 97, 47.5%). Prospective or retrospective ECG synchronization was applied as appropriate according to heart rate and clinical conditions. Oral metoprolol (25 mg) was administered before CCTA when additional heart-rate control was required. The scan range extended from the carina to 1 cm below the cardiac apex. Acquisition settings were individualized according to body habitus, with adjustment of tube voltage and automatic modulation of tube current. For coronary enhancement, iodinated contrast medium (370 mg I/mL) was delivered through an antecubital vein at a weight-adjusted dose of 1.0 mL/kg and an injection rate of 4–6 mL/s, followed by a 30–50 mL saline flush. Bolus tracking was performed with the region of interest positioned in the ascending aorta; once attenuation reached 100 HU, image acquisition was initiated after a 6-s delay. Reconstructed images were generated at a slice thickness and interval of 0.5–0.625 mm.

### 2.3. CCTA Image Analysis

Quantitative plaque assessment was performed on anonymized CCTA datasets using a semi-automated software package (uAI-CT Coronary Plaque, United Imaging, Shanghai, China). All software-derived contours of the coronary lumen, vessel wall, and plaque were visually verified and manually adjusted when necessary. Two radiologists, each with more than 5 years of experience in CCTA interpretation, independently reviewed the analyses; unresolved differences were referred to a senior radiologist for consensus. Plaque morphology was characterized by the presence of four established qualitative features: positive remodeling, low-attenuation plaque, napkin-ring sign, and spotty calcification [17]. The quantitative measurements comprised lesion length (LL), plaque volume (PV), vessel volume (VV), minimal lumen area (MLA), maximum diameter stenosis (MDS), maximum area stenosis (MAS), volumes of individual plaque components, remodeling index (RI), and eccentricity index (EI). Total plaque burden was derived as the percentage of PV relative to VV, whereas noncalcified plaque volume was obtained by combining the fibrous and lipid-rich components. Software-derived boundary values (e.g., MLA of 0 mm^2^ or MDS/MAS of 100%) were retained as quantitative outputs of the plaque-analysis software and were not considered indicative of true total or subtotal coronary occlusion. All measurements were performed on a vessel-specific basis for the left anterior descending (LAD), left circumflex (LCX), and right coronary artery (RCA).

### 2.4. ICA and FFR Assessment

ICA and FFR assessment were performed by experienced interventional cardiologists. A pressure wire was advanced distal to the target lesion, and resting Pd/Pa was recorded before FFR measurement during maximal hyperemia induced by intravenous infusion of adenosine triphosphate (ATP). Vessel-level ischemia was defined as FFR ≤ 0.80, and patients with at least one ischemic vessel were classified as ischemic. All vessels included in the analysis had an invasively measured FFR value. Detailed procedural protocols are provided in Appendix A.

### 2.5. Feature Selection

Candidate predictors were selected separately for each coronary artery. In the LAD and RCA cohorts, LASSO regression with five-fold cross-validation was used to identify relevant plaque features. For the smaller LCX cohort, Boruta with XGBoost as the base estimator was applied to accommodate potentially nonlinear feature relationships. In the primary five-fold cross-validation analysis, preprocessing, feature selection, and model fitting were performed entirely within each training fold, with the corresponding held-out fold used exclusively for model evaluation. Model stability was further assessed by bootstrap resampling. For LAD and RCA, feature selection was repeated within each bootstrap sample to quantify selection frequency and assess model performance. For LCX, Boruta feature selection was also repeated within each bootstrap training sample, followed by XGBoost model fitting and evaluation on the corresponding out-of-bag samples. To evaluate whether the vessel-specific findings were dependent on the use of different feature-selection and modeling algorithms, a sensitivity analysis was performed using a uniform LASSO-based feature-selection and logistic regression framework across all three coronary territories, following the same fold-wise cross-validation procedure.

### 2.6. Incremental Value Analysis

The incremental contribution of plaque characteristics was assessed using two vessel-specific logistic regression models. Model 1 comprised age, sex, hypertension, diabetes mellitus, and severe stenosis (≥70%). Model 2 included all Model 1 variables together with the plaque features retained by vessel-specific feature selection. Both models were evaluated using five-fold cross-validation. For both models, predicted probabilities were generated exclusively for the held-out fold and subsequently combined across the five folds to obtain out-of-fold predictions for all vessels. Areas under the receiver operating characteristic curve (AUC), ΔAUC, continuous net reclassification improvement (NRI), and integrated discrimination improvement (IDI) were calculated from these paired out-of-fold predictions.

### 2.7. Statistical Analysis

Continuous variables are summarized as mean ± SD or median (IQR), as appropriate, and categorical variables as n (%). Between-group differences were examined using the independent-samples t test, Mann–Whitney U test, or χ^2^ test, as appropriate. Intraobserver and interobserver reproducibility of quantitative CCTA-derived plaque measurements was assessed in 50 randomly selected patients. For intraobserver reproducibility, the same observer repeated the measurements after a one-week interval. For interobserver reproducibility, the measurements were independently performed by two observers. Intraclass correlation coefficients (ICCs) with 95% confidence intervals (CIs) were calculated using a two-way random-effects, single-measurement, absolute-agreement model. Vessel-specific predictive analyses were conducted separately for the LAD, LCX, and RCA, with at most one vessel per patient within each artery-specific dataset, although patients could contribute vessels to different coronary territories. For the incremental value analysis, ΔAUC, continuous NRI, and IDI were calculated. Their 95% CIs and two-sided *p* values were estimated using 1000 bootstrap resamples. All analyses were performed in Python 3.12, with statistical significance defined as a two-sided *p* < 0.05.

## 3. Results

### 3.1. Baseline Characteristics

Among the 204 included patients, 119 were assigned to the ischemic group and 85 to the non-ischemic group; together, they contributed 289 vessels (161 LAD, 55 LCX, and 73 RCA). Baseline characteristics were broadly similar between groups, with significant differences observed for sex, height and weight (Table 1). The median interval between CCTA and ICA was 13 days (IQR, 9–27 days).

### 3.2. Plaque Characteristics of Different Vascular Groups

Plaque characteristics differed between ischemic and non-ischemic vessels, with distinct patterns across coronary arteries. In the LAD, ischemia was associated with longer lesions, smaller MLA, more severe stenosis, and greater calcified and fibrous plaque burden (all *p* < 0.05). In the LCX, ischemic vessels exhibited longer lesions, smaller MLA, greater MDS and MAS, and higher total, calcified, and fibrotic plaque burden (all *p* < 0.05). In the RCA, ischemic vessels exhibited smaller MLA, more severe stenosis, and greater lipid and fibrous-fatty plaque burden (all *p* < 0.05). The eccentricity index was significantly lower in ischemic LCX and RCA vessels (both *p* < 0.05), whereas the napkin-ring sign was more frequent in ischemic LAD and RCA vessels (both *p* < 0.05). Comparisons of plaque characteristics according to ischemic status are presented separately for the LAD, LCX, and RCA in Table 2 and for all vessels combined in Appendix B Table A1. In the overall vessel cohort, FFR ranged from 0.58 to 1.02, with a median of 0.80 (IQR, 0.74–0.86). Of the 289 vessels, 151 (52.2%) had an FFR ≤ 0.80, whereas 138 (47.8%) had an FFR > 0.80.

### 3.3. Diagnostic Performance of Vessel-Specific Models

**Figure 2:**
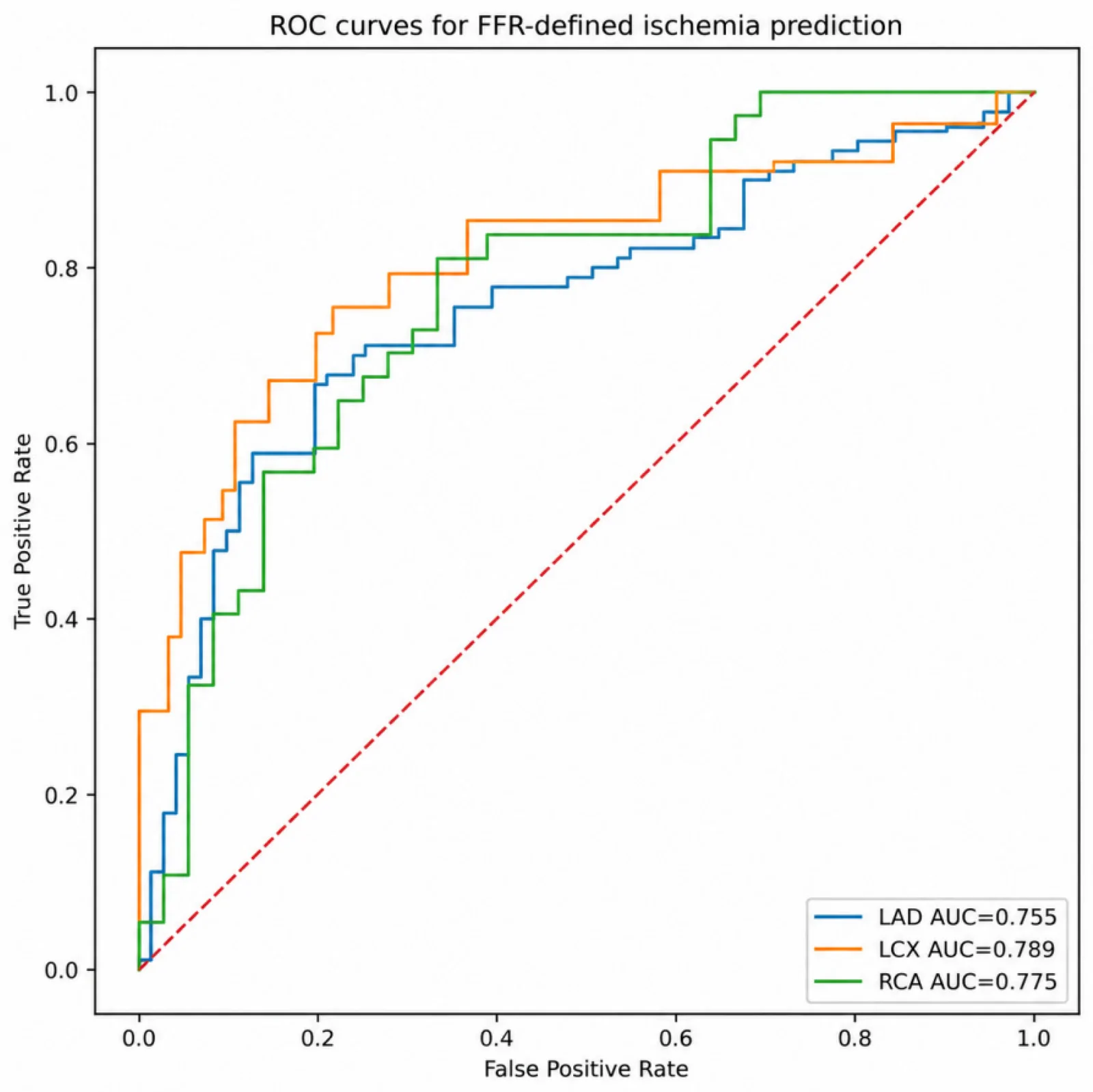
Receiver operating characteristic curves of the artery-specific models for identifying vessels with FFR ≤ 0.80. The red dashed line indicates the reference line for no discriminative ability (AUC = 0.5).

**Figure 3:**
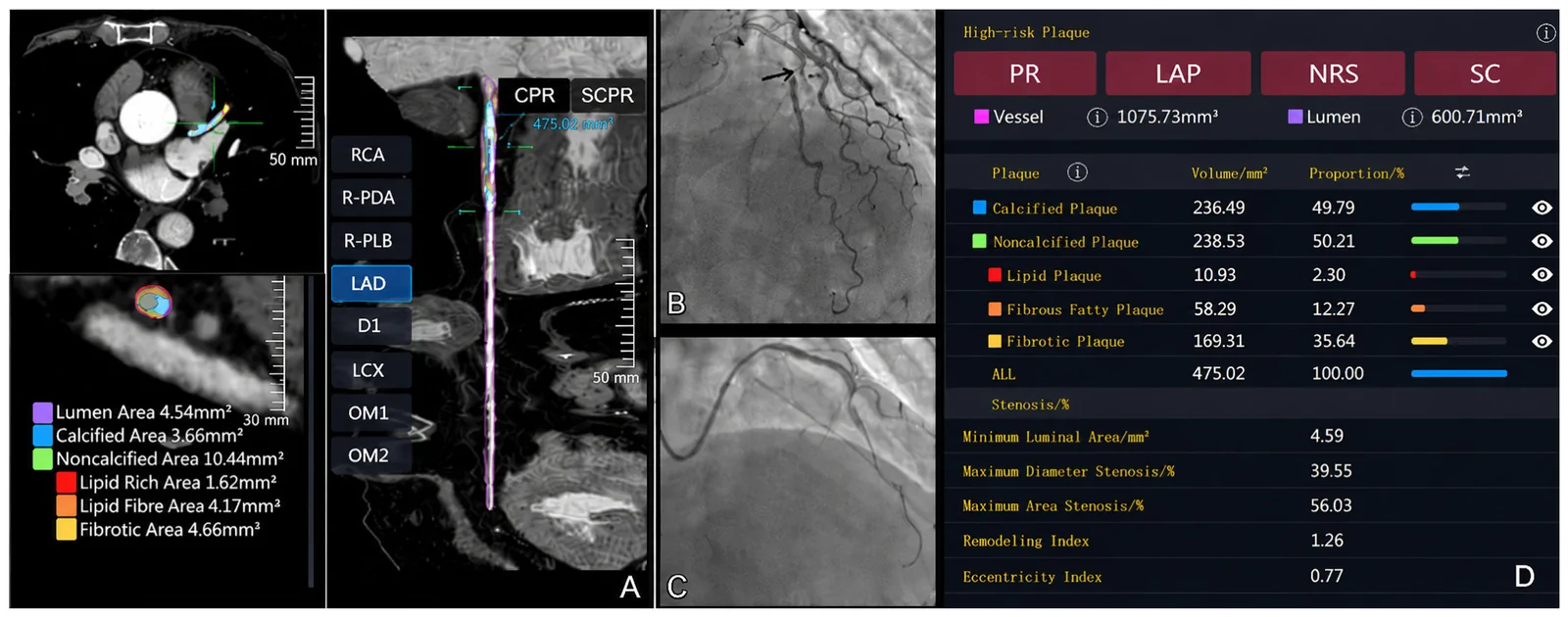
Multimodality assessment of a functionally significant LAD lesion in a 58-year-old woman. CCTA (**A**,**D**) demonstrated a single target lesion containing calcified plaque in the proximal-to-mid LAD, with a reduced minimum luminal area and increased plaque burden. Invasive coronary angiography (**B**) demonstrated 70–80% stenosis of the corresponding lesion. The black arrow indicates the corresponding lesion on invasive coronary angiography. Physiological assessment yielded an FFR of 0.73, and the lesion was subsequently treated with stent implantation (**C**).

**Figure A1:**
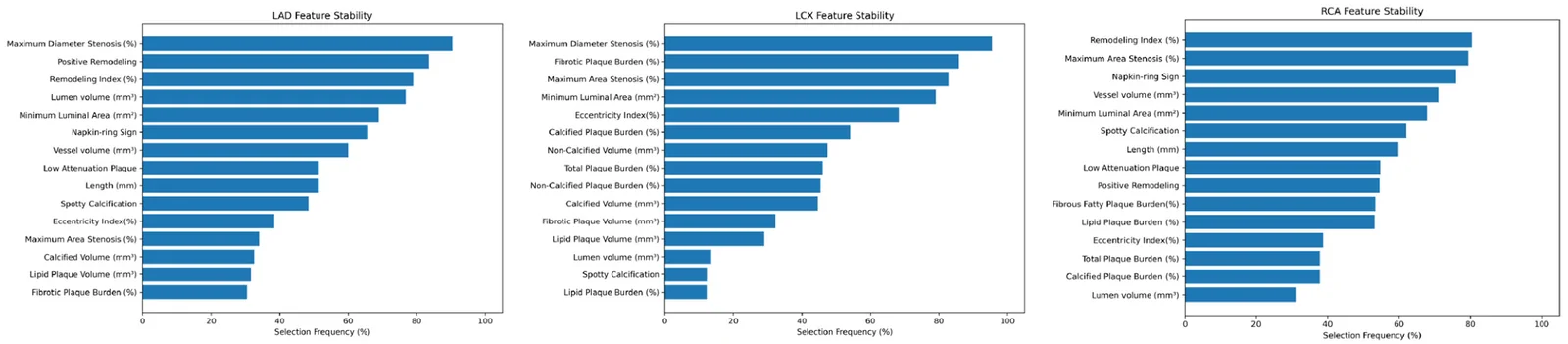
Stability of plaque feature selection across bootstrap resampling in LAD, LCX, and RCA vessels.

The features retained for ischemia prediction varied across coronary territories. The plaque features selected for ischemia prediction varied across coronary territories, with stenosis- and remodeling-related features predominating in the LAD, lesion geometry and stenosis-related features in the LCX, and remodeling and high-risk plaque characteristics in the RCA. Repeated bootstrap analyses supported the consistency of these vessel-dependent feature patterns (Appendix B Figure A1). After correction for optimism, the corresponding AUCs were 0.755 (95% CI, 0.682–0.826), 0.789 (95% CI, 0.656–0.905), and 0.775 (95% CI, 0.660–0.877) for the LAD, LCX, and RCA, respectively (Table 3; Figure 2). Figure 3 illustrates a representative LAD case. In the sensitivity analysis using a uniform LASSO-logistic regression framework across all three coronary territories, discriminatory performance remained broadly consistent with that of the primary analysis, with out-of-fold AUCs of 0.761 (95% CI, 0.684–0.834) for the LAD, 0.733 (95% CI, 0.579–0.868) for the LCX, and 0.777 (95% CI, 0.663–0.877) for the RCA (Appendix B Table A2). Differences in repeatedly selected plaque features across coronary territories were also retained.

### 3.4. Incremental Predictive Value for Ischemia

**Figure 4:**
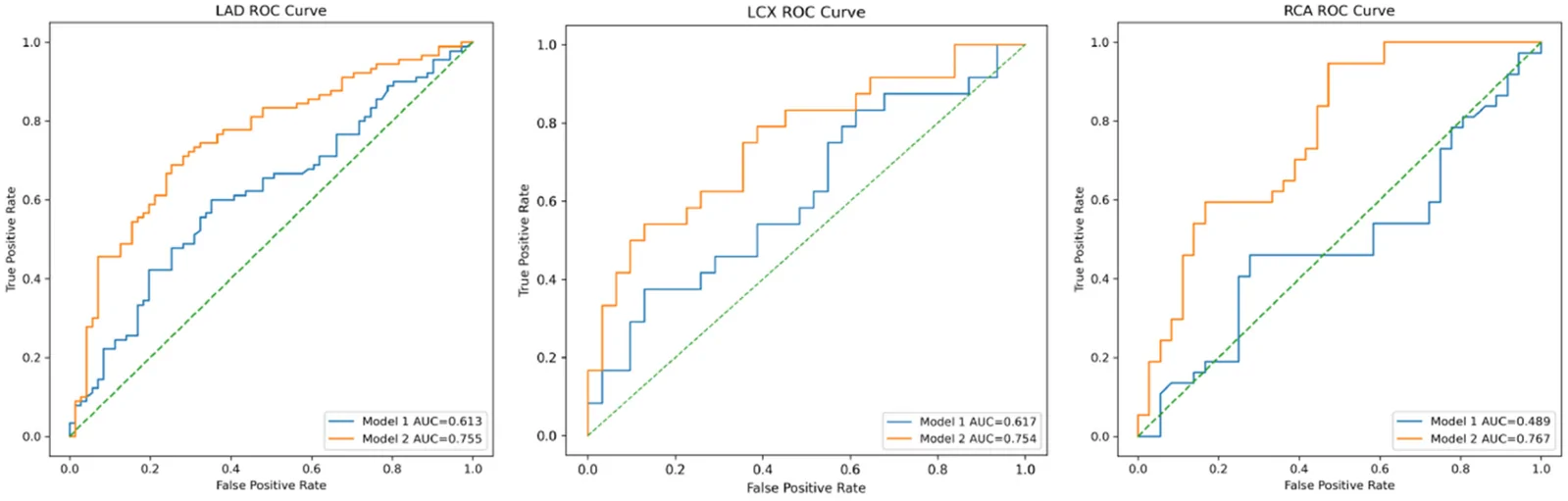
Artery-specific comparison of Model 1 and Model 2. Model 1 was constructed using age, sex, hypertension, diabetes mellitus, and maximum diameter stenosis (≥70% vs. <70%), while Model 2 further included the plaque features selected separately for each coronary artery. The green dashed line indicates the reference line for no discriminative ability (AUC = 0.5).

Model 2 outperformed Model 1 in all three coronary arteries after incorporation of the selected plaque features (Table 4; Figure 4). Based on paired out-of-fold predictions, the increase in AUC was greatest for the RCA (ΔAUC = 0.279), followed by the LAD (ΔAUC = 0.142) and LCX (ΔAUC = 0.137). NRI and IDI also indicated improved reclassification and discrimination across the vessel-specific models, with statistical significance maintained for all comparisons (*p* < 0.05).

Model 1 included age, sex, hypertension, diabetes mellitus, and maximum diameter stenosis (≥70% vs. <70%); Model 2 additionally incorporated the plaque features selected for each coronary artery. ΔAUC represents the difference in AUC between Model 2 and Model 1. AUC, ΔAUC, continuous NRI, and IDI were calculated from paired out-of-fold predictions generated by five-fold cross-validation. Confidence intervals and *p* values for incremental performance measures were estimated using 1000 bootstrap resamples. Abbreviations: AUC, area under the receiver operating characteristic curve; CI, confidence interval; NRI, net reclassification improvement; IDI, integrated discrimination improvement; LAD, left anterior descending artery; LCX, left circumflex artery; RCA, right coronary artery.

### 3.5. Plaque Analysis Reproducibility

Quantitative plaque measurements demonstrated excellent intraobserver and interobserver reproducibility, with ICCs ranging from 0.950 to 0.991 and from 0.921 to 0.984, respectively (Appendix B Table A3).

## 4. Discussion

This study identified vessel-dependent differences in the relationship between quantitative CCTA plaque characteristics and FFR-defined ischemia. The plaque features associated with ischemia varied among the LAD, LCX, and RCA, suggesting potential heterogeneity in plaque-ischemia associations across coronary arteries. Moreover, adding selected plaque features to clinical factors and stenosis severity improved predictive performance, although the extent of improvement differed across coronary arteries. These findings highlight the potential value of quantitative plaque assessment as an adjunct to conventional anatomical evaluation.

In agreement with previous studies, vessels with FFR-defined ischemia showed greater stenosis severity and a less favorable plaque profile [4,6,18,19]. This finding reinforces the limitation of luminal narrowing as a sole indicator of functional significance, which may also be influenced by lesion geometry, plaque composition, vascular remodeling, vessel caliber, and downstream myocardial demand [19,20,21,22]. Importantly, the associations between plaque-related features and FFR-defined ischemia appeared to vary among the LAD, LCX, and RCA, suggesting potential heterogeneity in plaque–ischemia relationships across coronary arteries. Bootstrap resampling provided an internal assessment of model performance variability; however, the reproducibility and generalizability of these findings require further evaluation in independent cohorts.

These findings extend recent vessel-specific quantitative CCTA studies. Nurmohamed et al. [4]. developed and externally validated a comprehensive AI-QCT model for ischemia prediction, whereas Belmonte et al. [20]. identified a single dominant plaque marker associated with ischemia for each major coronary artery. Lin et al. [6]. also investigated the predictive value of quantitative CCTA features using machine learning approaches, and Karlsberg et al. [23]. evaluated AI-guided quantitative CCTA for ischemia prediction in real-world practice. Building on these studies, the present analysis independently selected plaque characteristics for the LAD, LCX, and RCA and evaluated their incremental predictive value beyond a baseline model incorporating clinical risk factors and CCTA-derived stenosis severity. By considering plaque composition, plaque burden, vascular geometry, and qualitative high-risk plaque features, our findings suggest that the combinations of plaque characteristics associated with FFR-defined ischemia may differ among coronary arteries. These observations should be regarded as exploratory and require confirmation in independent cohorts.

Plaque vulnerability and hemodynamic significance represent different dimensions of coronary atherosclerosis. CCTA-derived high-risk plaque features have been linked to biological instability and adverse outcomes, with associations between plaque morphology and coronary physiology or clinical events also reported [21,22,24]. In our analysis, however, their contribution to FFR-defined ischemia varied among coronary arteries, even though features such as positive remodeling and the napkin-ring sign were retained in some vessel-specific models. This variability may reflect the greater dependence of flow limitation on lesion geometry, overall plaque burden, and their combined effects on coronary hemodynamics. Thus, high-risk plaque features may complement quantitative plaque measurements rather than serve as interchangeable markers of functional significance.

The incremental contribution of plaque characteristics was evident across all three coronary arteries, but varied in magnitude among vessels. This variability indicates that similar degrees of luminal narrowing do not necessarily imply comparable physiological effects. Incorporating plaque information may therefore provide a more complete anatomical characterization of lesions relevant to functional significance. Clinically, vessel-specific plaque assessment may complement conventional CCTA interpretation by helping identify lesions that warrant further functional evaluation, particularly when stenosis severity alone does not adequately reflect the suspected physiological significance.

FFR-CT provides a noninvasive estimate of coronary physiological significance, whereas quantitative CCTA plaque analysis characterizes plaque burden, composition, and morphology. Although both approaches may contribute to identifying functionally significant coronary disease, they provide different types of information and may be complementary. The present study focused on vessel-specific plaque characteristics using invasive FFR as the reference standard rather than directly evaluating FFR-CT. Therefore, our findings do not establish the comparative performance of plaque-based models and FFR-CT. Future studies incorporating both approaches are warranted to determine whether quantitative plaque characteristics provide incremental value beyond FFR-CT.

Several limitations should be acknowledged. First, this was a retrospective, single-center study without external validation, and differences in CT acquisition and reconstruction across scanner platforms may have affected plaque quantification, limiting the generalizability of the findings. Second, the relatively small sample size, particularly in the LCX subgroup, with a limited number of ischemic events relative to model complexity and a residual risk of overfitting, and potential within-patient correlation across coronary territories may limit the precision and interpretation of vessel-specific findings, which should be considered exploratory. Third, ICA referral and FFR measurement were clinically determined, introducing potential selection and verification bias. In addition, the exclusion of vessels with multiple discrete plaques proximal to the FFR measurement position and complex diffuse coronary disease may limit the generalizability of our findings to unselected CCTA populations and patients with more complex coronary artery disease. Fourth, the analysis focused on CCTA-derived plaque characteristics and did not incorporate other emerging imaging biomarkers, such as pericoronary adipose tissue [25], which may provide additional information relevant to ischemia. Furthermore, FFR-CT measurements were not available, precluding direct comparison of the proposed plaque-based models with this established noninvasive functional assessment approach. Prospective multicenter validation and direct comparisons with FFR-CT are warranted to establish the potential clinical utility of these findings.

## 5. Conclusions

The relationship between coronary plaque phenotype and functional significance appeared to differ among the LAD, LCX, and RCA. Incorporating artery-specific plaque information into clinical and stenosis-based assessment improved the prediction of FFR-defined ischemia, particularly in the LAD and RCA. These exploratory findings are hypothesis-generating and require validation in larger, independent cohorts.

## Figures and Tables

**Table 1:** Clinical and demographic characteristics according to FFR-defined ischemic status.

Study Population	Patient				
	FFR > 0.8 (*n* = 85)		FFR ≤ 0.8 (*n* = 119)		*p*-Value
Age (years)	64.00 (59.00–69.00)		63.00 (58.00–69.00)		0.400
Female (n (%))	35 (41.2%)		25 (21.0%)		0.002
Height (cm)	165.60 ± 7.76		168.99 ± 7.84		0.008
Weight (kg)	67.60 (60.00, 72.00)		72.50 (65.00, 79.75)		0.001
BMI (kg/m^2^)	24.16 (22.46, 26.40)		25.37 (23.50, 27.03)		0.073
Basic Disease					
Hypertension (n (%))	64 (75.3%)		85 (71.4%)		0.540
Diabetes Mellitus (n (%))	38 (44.7%)		44 (37.0%)		0.267
Dyslipidemia (n (%))	68 (80.0%)		92 (77.3%)		0.645
Previous History					
History of CAD (n (%))	20 (23.5%)		36 (30.3%)		0.289
Smoking (n (%))	35 (41.2%)		64 (53.8%)		0.076
Drinking (n (%))	32 (37.6%)		55 (46.2%)		0.222
NYHA functional class (n (%))	I	56 (65.9%)	I	77 (64.7%)	0.656
	II	27 (31.8%)	II	41 (34.5%)	
	III	2 (2.3%)	III	1 (0.8%)	
Other Cardiovascular Diseases (n (%))	Valvular heart disease	2 (2.3%)	Valvular heart disease	2 (1.7%)	/
	Arrhythmias	8 (9.4%)	Arrhythmias	13 (10.9%)	

CAD, coronary artery disease; BMI, body mass index; and NYHA, New York Heart Association.

**Table 2:** Vessel-specific plaque characteristics stratified by FFR status.

	LAD			LCX			RCA		
	FFR > 0.8 (*n* = 71)	FFR ≤ 0.8 (*n* = 90)	*p*-Value	FFR > 0.8 (*n* = 31)	FFR ≤ 0.8 (*n* = 24)	*p*-Value	FFR > 0.8 (*n* = 36)	FFR ≤ 0.8 (*n* = 37)	*p*-Value
LL (mm)	24.90 (16.95–39.60)	36.60 (23.40–47.62)	0.004	9.90 (9.00–14.18)	22.35 (14.32–31.50)	0.003	18.90 (13.05–34.05)	27.30 (17.10–52.50)	0.102
LV (mm^3^)	786.88 (684.27–920.60)	676.50 (562.14–773.97)	<0.001	367.44 (314.50–415.61)	350.15 (251.15–426.22)	0.157	780.12 ± 351.78	628.41 ± 237.86	0.035
VV (mm^3^)	991.34 (857.15–1209.95)	936.34 (829.75–1066.07)	0.086	467.35 (373.77–636.35)	483.46 (363.23–728.31)	0.849	1077.86 ± 536.45	919.88 ± 350.17	0.143
MLA (mm^2^)	1.89 (1.53–2.56)	1.08 (0.16–1.62)	<0.001	2.25 (1.62–3.58)	0.99 (0.00–1.82)	<0.001	2.43 (1.64–3.46)	1.35 (0.81–1.89)	<0.001
MDS (%)	61.65 (53.40–67.05)	72.93 (65.18–96.12)	<0.001	48.54 (35.35–59.26)	68.53 (55.13–100.00)	<0.001	56.27 (43.56–63.64)	65.46 (58.98–73.97)	<0.001
MAS (%)	78.14 (69.36–83.02)	87.66 (78.81–98.49)	<0.001	63.52 (50.12–75.42)	85.06 (68.19–100.00)	<0.001	70.53 ± 16.28	81.89 ± 10.41	<0.001
Calcified Volume (mm^3^)	21.76 (1.64–95.51)	65.53 (11.20–118.55)	0.011	2.64 (0.23–7.08)	17.94 (2.24–86.48)	0.007	15.34 (2.42–45.17)	35.06 (2.00–59.62)	0.543
Non-Calcified Volume (mm^3^)	115.20 (41.82–171.36)	133.28 (66.34–231.77)	0.053	21.20 (7.73–41.01)	41.34 (14.24–93.32)	0.052	82.26 (23.72–156.34)	150.49 (34.52–252.48)	0.210
Lipid Plaque Volume (mm^3^)	7.45 (1.86–15.58)	9.62 (3.65–20.74)	0.082	0.63 (0.00–1.49)	0.79 (0.00–5.83)	0.315	3.27 (0.21–15.08)	9.40 (1.80–21.42)	0.088
Fibrous Fatty Volume (mm^3^)	40.25 (12.98–68.27)	49.64 (21.09–81.66)	0.121	6.63 (0.91–15.98)	9.53 (1.83–38.24)	0.385	34.51 (6.85–67.44)	52.36 (13.02–99.13)	0.116
Fibrotic Plaque Volume (mm^3^)	47.64 (17.43–80.92)	72.15 (38.57–118.54)	0.014	9.83 (3.80–16.09)	27.96 (6.09–61.47)	0.044	31.86 (13.88–97.61)	56.74 (19.43–128.18)	0.399
Quantitative Plaque Burden									
TPB (%)	17.27 (7.75–25.25)	25.71 (14.38–33.73)	<0.001	5.65 (3.65–8.47)	10.29 (6.67–27.93)	0.015	10.93 (6.55–20.26)	16.84 (7.72–29.33)	0.184
CPB (%)	2.44 (0.18–8.96)	6.67 (1.30–13.21)	0.008	0.51 (0.05–1.88)	7.15 (0.78–20.13)	0.002	1.53 (0.34–6.31)	4.03 (0.30–7.97)	0.364
NCPB (%)	11.76 (4.83–16.06)	14.18 (8.18–22.31)	0.004	4.26 (1.74–6.23)	7.91 (3.10–11.54)	0.084	8.25 (3.54–15.65)	14.04 (5.27–20.53)	0.124
LPB (%)	0.63 (0.22–1.64)	0.93 (0.45–2.13)	0.036	0.12 (0.00–0.27)	0.28 (0.02–0.88)	0.130	0.35 (0.02–1.27)	1.18 (0.23–2.42)	0.032
FFPB (%)	3.81 (1.42–6.37)	5.33 (2.49–8.00)	0.027	1.33 (0.28–2.90)	2.47 (0.69–5.05)	0.177	3.56 (0.70–5.88)	5.62 (2.25–10.03)	0.015
FPB (%)	5.04 (2.32–8.66)	7.88 (4.35–12.02)	0.001	1.88 (1.13–3.11)	5.67 (2.11–13.42)	0.003	4.23 (1.91–8.34)	6.29 (2.93–12.99)	0.126
Vulnerability Features									
Remodeling Index	1.36 (1.20–2.04)	1.36 (1.24–1.65)	0.842	1.16 (1.06–1.34)	1.20 (1.09–1.49)	0.230	1.26 (1.14–2.01)	1.30 (1.21–1.47)	0.484
Eccentricity Index	1.00 (0.61–1.00)	0.93 (0.72–1.00)	0.869	1.00 (0.78–1.00)	0.85 (0.74–1.00)	0.042	0.92 (0.64–1.00)	0.64 (0.55–0.77)	<0.001
Positive Remodeling	48 (67.6%)	74 (82.2%)	0.032	15 (48.4%)	12 (50.0%)	0.906	22 (61.1%)	28 (75.7%)	0.180
Low Attenuation Plaque	62 (87.3%)	84 (93.3%)	0.193	21 (67.7%)	18 (75.0%)	0.557	27 (75.0%)	33 (89.2%)	0.113
Napkin-ring Sign	18 (25.4%)	41 (45.6%)	0.008	3 (9.7%)	7 (29.2%)	0.063	8 (22.2%)	18 (48.6%)	0.018
Spotty Calcification	27 (38.0%)	45 (50.0%)	0.129	4 (12.9%)	8 (33.3%)	0.069	10 (27.8%)	12 (32.4%)	0.665

Continuous variables are presented as mean ± standard deviation (SD) for normally distributed data or median (interquartile range, IQR) for non-normally distributed data, as appropriate. Categorical variables are presented as number (percentage). The between-group comparisons were exploratory, and *p* values were not adjusted for multiple testing. These results should therefore be interpreted as descriptive rather than confirmatory. LAD, left anterior descending artery; LCX, left circumflex artery; RCA, right coronary artery; FFR, fractional flow reserve; LL, lesion length; LV, lumen volume; VV, vessel volume; MLA, minimum luminal area; MDS, maximum diameter stenosis; MAS, maximum area stenosis; TPB, total plaque burden; CPB, calcified plaque burden; NCPB, non-calcified plaque burden; LPB, lipid plaque burden; FFPB, fibrous fatty plaque burden; and FPB, fibrotic plaque burden.

**Table 3:** Diagnostic performance of vessel-specific plaque-based prediction models for FFR-defined myocardial ischemia.

Vessel	AUC	AUC 95%CI	Accuracy	Sensitivity	Specificity
LAD	0.755	0.682–0.826	0.727	0.667	0.803
LCX	0.789	0.656–0.905	0.764	0.708	0.806
RCA	0.775	0.661–0.877	0.740	0.811	0.667

**Table 4:** Comparison of Model 1 and Model 2 performance across individual coronary arteries.

Vessel	Model 1 AUC (95%CI)	Model 2 AUC (95%CI)	ΔAUC (95%CI)	*p* Value	NRI (95%CI)	*p* Value	IDI (95%CI)	*p* Value
LAD	0.613 (0.527–0.699)	0.755 (0.680–0.828)	0.142 (0.061–0.217)	0.002	0.736 (0.449–1.016)	<0.001	0.163 (0.1004–0.220)	<0.001
LCX	0.617 (0.461–0.769)	0.754 (0.609–0.884)	0.137 (0.006–0.290	<0.001	0.651 (0.133–1.157)	0.014	0.169 (0.057–0.276)	0.002
RCA	0.489 (0.364–0.628)	0.767 (0.651–0.868)	0.279 (0.144–0.408)	<0.001	0.955 (0.564–1.342)	<0.001	0.276 (0.163–0.380)	<0.001

## Data Availability

The datasets used and/or analyzed during the current study are available from the corresponding author upon reasonable request.

## Abbreviations

The following abbreviations are used in this manuscript:

AUC	Area under the ROC curve
BMI	Body mass index
CAD	coronary artery disease
CCTA	coronary computed tomography angiography
CPB	Calcified plaque burden
FFR-CT	CCTA-derived FFR
EI	eccentricity index
FFPB	Fibrous fatty plaque burden
FFR	fractional flow reserve
FPB	Fibrotic plaque burden
ICA	invasive coronary angiography
ICC	intraclass correlation coefficient
IDI	Integrated discrimination improvement
IQR	Interquartile range
LAD	left anterior descending
LAP	Low-attenuation plaque
LASSO	least absolute shrinkage and selection operator
LCX	left circumflex
LL	Lesion length
LPB	Lipid plaque burden
LV	Lumen volume
MAS	maximum area stenosis
MDS	maximum diameter stenosis
MLA	minimal lumen area
NCPB	Noncalcified plaque burden
NRI	Net reclassification improvement
NYHA	New York Heart Association
PB	Plaque burden
Pd/Pa	Distal coronary pressure-to-aortic pressure ratio
PV	plaque volume
RCA	right coronary artery
RI	remodeling index
ROC	Receiver operating characteristic curve
TPB	Total plaque burden
VV	vessel volume
XGboost	Extreme gradient boosting

## Appendix A

ICA was undertaken by experienced interventional cardiologists through right radial access. Following local infiltration with 2% lidocaine, a 6F arterial sheath was inserted, through which heparin (3000 U) and nitroglycerin (200 μg) were administered before angiographic imaging. The decision to perform FFR measurement was made by the treating interventional cardiologists in accordance with routine clinical practice, taking into account angiographic stenosis severity and the overall clinical context. FFR was not systematically performed in all coronary vessels. For FFR measurement, a guiding catheter was engaged at the coronary ostium and a pressure-wire sensor was positioned distal to the target lesion. Resting Pd/Pa was recorded, followed by FFR measurement during maximal hyperemia induced by intravenous infusion of adenosine triphosphate (ATP) at 140–180 μg/kg/min. Measurements were obtained after stable maximal hyperemia was achieved. Pressure drift was checked after measurement, and pressure-wire pullback was performed. Two physicians independently evaluated the FFR measurements, and any discrepancy was adjudicated by a senior reader. FFR measurements obtained in side branches were assigned to their corresponding major coronary territories (D1/D2 to the LAD, OM to the LCX, and PDA/PLB to the RCA). In all cases, the analyzed CCTA plaque was anatomically matched to the FFR-interrogated lesion, and only vessels with a single identifiable plaque proximal to the FFR measurement position were included.

## Appendix B

**Table A1:** Vessel-level differences in qualitative and quantitative plaque features. Continuous data are reported as mean ± standard deviation or median (interquartile range), as appropriate; categorical data are expressed as number (percentage). Abbreviations: LL, lesion length; LV, lumen volume; VV, vessel volume; MLA, minimum luminal area; MDS, maximum diameter stenosis; MAS, maximum area stenosis; TPB, total plaque burden; CPB, calcified plaque burden; NCPB, non-calcified plaque burden; LPB, lipid plaque burden; FFPB, fibrous fatty plaque burden; FPB, fibrotic plaque burden; and FFR, fractional flow reserve.

	Vessel		*p*-Value
	FFR > 0.8 (*n* = 138)	FFR ≤ 0.8 (*n* = 151)	
LL (mm)	19.20 (10.95–34.65)	32.70 (17.55–46.80)	<0.001
LV (mm^3^)	731.55 (536.67–902.31)	614.60 (457.88–765.86)	0.001
VV (mm^3^)	931.17 (633.92–1161.55)	882.19 (671.35–1056.93)	0.353
MLA (mm^2^)	2.16 (1.53–2.88)	1.17 (0.72–1.71)	<0.001
MDS (%)	58.47 (47.60–65.43)	70.02 (59.92–83.20)	<0.001
MAS (%)	73.02 (61.80–82.05)	86.21 (76.27–93.57)	<0.001
Calcified Volume (mm^3^)	9.63 (1.60–46.21)	46.15 (4.08–107.23)	<0.001
Non-Calcified Volume (mm^3^)	67.52 (20.79–149.54)	114.84 (52.12–219.67)	<0.001
Lipid Plaque Volume (mm^3^)	3.06 (0.57–12.89)	7.79 (2.23–19.63)	0.001
Fibrous Fatty Volume (mm^3^)	28.75 (6.44–61.00)	43.95 (12.95–76.46)	0.004
Fibrotic Plaque Volume (mm^3^)	32.23 (11.61–71.02)	60.17 (26.13–115.73)	<0.001
Quantitative Plaque Burden			
TPB (%)	10.47 (5.43–22.01)	22.02 (10.29–32.88)	<0.001
CPB (%)	1.54 (0.18–6.88)	5.79 (0.69–12.82)	<0.001
NCPB (%)	7.99 (3.41–15.24)	12.81 (7.11–20.49)	<0.001
LPB (%)	0.40 (0.08–1.24)	0.88 (0.31–2.09)	<0.001
FFPB (%)	3.07 (0.89–5.75)	4.87 (2.10–8.22)	<0.001
FPB (%)	3.71 (1.82–7.37)	7.41 (3.43–12.66)	<0.001
Vulnerability Features			
Remodeling Index	1.28 (1.13–1.77)	1.33 (1.21–1.56)	0.396
Eccentricity Index	1.00 (0.64–1.00)	0.81 (0.62–1.00)	0.015
Positive Remodeling (%)	85 (61.6%)	114 (75.5%)	0.011
Low Attenuation Plaque (%)	110 (79.7%)	135 (89.4%)	0.022
Napkin-ring Sign (%)	30 (21.7%)	66 (43.7%)	<0.001
Spotty Calcification (%)	41 (29.7%)	65 (43.0%)	0.019
FFR value	0.86 (0.83–0.91)	0.74 (0.71–0.76)	<0.001

**Table A2:** Sensitivity analysis using a uniform LASSO-logistic regression framework across coronary territories.

Coronary Artery	N	Repeatedly Selected Plaque Features *	AUC (95%CI)
LAD	161	Lumen volume; MDS; remodeling index; positive remodeling; napkin-ring sign; vessel volume; MLA	0.761 (0.684–0.834)
LCX	55	MDS; fibrotic plaque burden; calcified plaque burden	0.733 (0.579–0.868)
RCA	73	Fibrous fatty plaque burden; MAS; remodeling index; MLA; vessel volume; napkin-ring sign	0.777 (0.663–0.877)

* Repeatedly selected plaque features were defined as variables selected in ≥3 of the five cross-validation folds. Preprocessing, feature selection, and model fitting were performed entirely within each training fold. AUCs were calculated from pooled out-of-fold predictions, and 95% CIs were estimated using bootstrap resampling. Abbreviations: AUC, area under the receiver operating characteristic curve; LAD, left anterior descending artery; LCX, left circumflex artery; RCA, right coronary artery; MDS, maximum diameter stenosis; MLA, minimum luminal area; and MAS, maximum area stenosis.

**Table A3:** Patient-level intraobserver and interobserver reproducibility of CCTA-derived quantitative plaque measurements.

Plaque Parameter	Intraobserver ICC (95% CI)	Interobserver ICC (95% CI)
LL (mm)	0.979 (0.963–0.989)	0.958 (0.927–0.976)
LV (mm^3^)	0.987 (0.977–0.993)	0.978 (0.961–0.988)
VV (mm^3^)	0.989 (0.980–0.994)	0.981 (0.966–0.989)
MLA (mm^2^)	0.973 (0.952–0.985)	0.946 (0.906–0.969)
MDS (%)	0.968 (0.944–0.982)	0.938 (0.892–0.965)
MAS (%)	0.965 (0.938–0.980)	0.932 (0.882–0.962)
Noncalcified plaque volume (mm^3^)	0.982 (0.967–0.990)	0.963 (0.935–0.979)
Calcified plaque volume (mm^3^)	0.976 (0.957–0.987)	0.952 (0.917–0.973)
Lipid plaque volume (mm^3^)	0.950 (0.912–0.972)	0.921 (0.865–0.955)
TPB (%)	0.986 (0.975–0.992)	0.976 (0.957–0.986)
CPB (%)	0.974 (0.954–0.985)	0.949 (0.911–0.971)
NCPB (%)	0.981 (0.966–0.989)	0.961 (0.932–0.978)
LPB (%)	0.953 (0.917–0.974)	0.925 (0.872–0.957)
FFPB (%)	0.971 (0.949–0.983)	0.943 (0.901–0.967)
FPB (%)	0.977 (0.959–0.987)	0.955 (0.922–0.974)

Intraobserver and interobserver reproducibility were assessed at the patient level. ICC estimates and their corresponding 95% confidence intervals were calculated separately for each quantitative parameter using a two-way random-effects, single-measurement, absolute-agreement model. ICC, intraclass correlation coefficient; CI, confidence interval. LL, lesion length; LV, lumen volume; VV, vessel volume; MLA, minimum lumen area; MDS, maximum diameter stenosis; MAS, maximum area stenosis; TPB, total plaque burden; CPB, calcified plaque burden; NCPB, noncalcified plaque burden; LPB, Lipid plaque burden; FFPB, fibrofatty plaque burden; and FPB, fibrous plaque burden.
